# Recent Advances in Self-Supported Layered Double Hydroxides for Oxygen Evolution Reaction

**DOI:** 10.34133/2020/3976278

**Published:** 2020-02-19

**Authors:** Libo Wu, Luo Yu, Xin Xiao, Fanghao Zhang, Shaowei Song, Shuo Chen, Zhifeng Ren

**Affiliations:** ^1^Department of Physics and Texas Center for Superconductivity (TcSUH), University of Houston, Houston, TX 77204, USA; ^2^Materials Science and Engineering Program, University of Houston, Houston, TX 77204, USA; ^3^College of Physical Science and Technology, Central China Normal University, Wuhan 430079, China; ^4^Wuhan National Laboratory for Optoelectronics, Huazhong University of Science and Technology, Wuhan 430074, China; ^5^Department of Chemistry, University of Houston, Houston, Texas 77204, USA

## Abstract

Electrochemical water splitting driven by clean and sustainable energy sources to produce hydrogen is an efficient and environmentally friendly energy conversion technology. Water splitting involves hydrogen evolution reaction (HER) and oxygen evolution reaction (OER), in which OER is the limiting factor and has attracted extensive research interest in the past few years. Conventional noble-metal-based OER electrocatalysts like IrO_2_ and RuO_2_ suffer from the limitations of high cost and scarce availability. Developing innovative alternative nonnoble metal electrocatalysts with high catalytic activity and long-term durability to boost the OER process remains a significant challenge. Among all of the candidates for OER catalysis, self-supported layered double hydroxides (LDHs) have emerged as one of the most promising types of electrocatalysts due to their unique layered structures and high electrocatalytic activity. In this review, we summarize the recent progress on self-supported LDHs and highlight their electrochemical catalytic performance. Specifically, synthesis methods, structural and compositional parameters, and influential factors for optimizing OER performance are discussed in detail. Finally, the remaining challenges facing the development of self-supported LDHs are discussed and perspectives on their potential for use in industrial hydrogen production through water splitting are provided to suggest future research directions.

## 1. Introduction

The emission of greenhouse gases and other environmental pollution issues related to the consumption of fossil fuels such as coal, petroleum, and natural gas has aroused intensive research interest in renewable energy technologies [1–4]. Hydrogen has been proved to be an ideal energy carrier for sustainable energy systems due to its zero-carbon emissions and high energy density [5–10]. Hydrogen production through electrochemical water splitting is a more efficient and “greener” technology compared with the traditional steam-reforming method used to obtain hydrogen from natural gas [11–13]. In particular, electrochemical water splitting can be driven by the electricity generated by clean and sustainable energy sources like solar and wind power, making it economically affordable and energy-saving [1, 14–16]. In general, the water splitting process is composed of two half reactions: hydrogen evolution reaction (HER) on the cathode and oxygen evolution reaction (OER) on the anode [17, 18]. It is widely acknowledged that the OER process, which has a large energy barrier, is more kinetically sluggish than HER, and thereby seriously limits the practical utilization of overall water splitting [19, 20]. Additionally, OER plays a crucial role in some other energy-storage and energy-conversion systems such as CO_2_ reduction, fuel cells, and metal–air batteries [21–24].

Electrocatalysts, which can reduce the overpotential required for water splitting by lowering the activation energy, have been widely employed to promote OER kinetics [6, 25]. Conventional precious-metal-based electrocatalysts like IrO_2_ and RuO_2_ exhibit excellent OER performance [26]. However, their high cost, scarce availability, and instability in alkaline media at large current density significantly impede the large-scale industrial applications of these noble-metal catalysts [27]. Thus, it is highly desirable to search for and develop non-noble-metal electrocatalysts that are cost-efficient and exhibit long-term stability to replace these expensive electrocatalysts. In the past few decades, significant progress has been made in fabricating novel alternative non-noble-metal-based electrocatalysts [17, 28]. Layered double hydroxides (LDHs) have emerged as among the most promising candidates for OER catalysis due to their unique layered structures and high electrocatalytic activity [27]. In general, LDH materials are brucite-like lamellar crystals composed of positive host layers and charge-balancing interlayers. All of the LDH materials can be classified by a general formula as M^2+^_1−*x*_M^3+^_*x*_(OH)_2_(A^*n*−^)_*x*/*n*_ · yH_2_O, in which M^2+^ and M^3+^ represent divalent and trivalent cations such as Ni^2+^, Co^2+^, Fe^2+^, Zn^2+^ and Fe^3+^, Al^3+^, Cr^3+^, and Mn^3+^, respectively, and A^*n*−^ is the charge-compensating interlayer anion such as CO_3_^2−^, NO_3_^−^, SO_4_^2−^, and Cl^−^ [27, 29–31]. Most LDH materials exhibit two-dimensional- (2D-) layered nanosheet structures, on which metal cations can be located [31]. Additionally, such 2D nanosheet structures make the cations and anions in the host layers and interlayers flexibly tunable, which offers more opportunities for fabricating novel LDH electrocatalysts [32].

Although LDH-based materials have been developed as promising OER electrocatalysts due to their various advantages, such as low cost, abundance, and favorable water adsorption, the poor durability and low intrinsic conductivity (10^−13^–10^−17^ S cm^−1^) of the powdery LDHs greatly hinder their electrocatalytic performance and practical application [33]. More importantly, a nonconductive polymer binder is needed to attach the LDH powder onto a conductive substrate for electrode preparation. On the one hand, using a nonconductive binder significantly increases the charge-transfer resistance of the electrode. On the other hand, the formation of strong bubbles during the OER process would damage the connection between the powdery LDH and the substrate, leading to catalyst collapse [34]. A self-supported electrocatalyst architecture with enhanced kinetics and stability is more attractive than the conventional-coated powdery form. Excellent work has been reported on self-supported LDHs grown *in situ* on conductive supports toward water splitting [35, 36]. In addition to their excellent charge-transfer ability, conventional supports like nickel foam (NF), copper foam (CF), and carbon cloth (CC) can provide a large surface area for active electrocatalysts to grow [10, 37]. Generally speaking, the rational growth of self-supported LDHs on substrates can accelerate the OER process based on the following aspects: (1) direct growth of LDH nanoarrays on conductive bones not only immobilizes the LDH's unique lamellar structure on the electrode surface but also promotes electron transport from the LDH to the substrate due to the intimate interfacial connection, (2) the large surface area of this open structure is beneficial to the contact between the electrode and the electrolyte media, which guarantees that the active sites effectively take part in the OER and avoid potential aggregation, and (3) the self-supported LDH nanoarrays with an ordered structure and open space are helpful for the gas-bubble release by providing smooth diffusion paths for gaseous products [34, 38, 39]. Compared with powdery catalysts, a self-supported LDH on a substrate can be directly utilized as an electrode for the electrochemical tests, while the former often requires the addition of an insulating polymeric binder and a multistep operation to assemble the electrode.

To date, there have been no relevant reviews focusing on the development of self-supported LDHs toward OER. Here, we summarize the recent advances in the development of self-supported LDH electrocatalysts and highlight their catalytic performance toward OER. This review begins with a basic introduction to the fundamental mechanisms and important evaluation parameters of OER, as well as the details for some synthetic methods typically used to fabricate self-supported LDHs. Next, different kinds of self-supported bimetallic and trimetallic LDHs, as well as their derivatives, are highlighted and discussed in detail. Finally, the remaining challenges and future outlook for the design and synthesis of novel self-supported LDH electrocatalysts are discussed to provide direction for further research.

## 2. Fundamentals of Oxygen Evolution Reaction

### 2.1. Principles of Oxygen Evolution Reaction

In broad terms, the OER process can be divided into three sequential steps: adsorption of water molecules onto the catalyst surface, then splitting of the water into molecular oxygen through a complicated process, and finally molecular oxygen release [40]. Since LDH materials can dissolve in acid conditions, they should ordinarily be employed in alkaline media (mainly aqueous KOH solution) for the OER process. The general reaction process of OER in alkaline media can be expressed as follows [32]:

(1) $$\begin{array}{c} {\text{OH}}^{-}{+}^{*}⟶{\text{OH}}^{*}+{\text{e}}^{-}\\ {\text{OH}}^{*}+{\text{OH}}^{-}⟶{\text{O}}^{*}+{\text{H}}_{2}\text{O}+{\text{e}}^{-}\\ {\text{OH}}^{-}+{\text{O}}^{*}⟶{\text{OOH}}^{*}+{\text{e}}^{-}\\ {\text{OOH}}^{*}+{\text{OH}}^{-}⟶{\text{O}}_{2}{+}^{*}+{\text{H}}_{2}\text{O}+{\text{e}}^{-} \end{array}$$

Here, ∗ represents the adsorption site on the electrocatalyst and O^∗^ and OH^∗^ denote adsorbed intermediates. Each equation above generates one electron, so the whole OER process in alkaline media includes a four-electron transfer. The catalytic activity of a specific OER process can be preliminarily evaluated by cyclic voltammetry (CV) and linear sweep voltammetry (LSV) testing. However, to fairly judge the OER performance of a given catalyst, some other crucial parameters, including overpotential (*η*), Tafel plot (*b*), exchange current density (*j*_0_), and turnover frequency (TOF), are necessary [41]. In the following, we will explain each of these parameters in more detail.

### 2.2. Overpotential

Among the four parameters mentioned above, overpotential (*η*) is the most important. In theory, the electrocatalytic reaction of OER can be driven by applying a voltage equal to the equilibrium hydrolysis potential (1.23 V *vs.* RHE). In reality, however, extra electrical energy (overpotential, *η*) is needed to surpass the kinetic barrier. According to the Nernst equation, for an electrocatalytic redox reaction, the applied potential can be expressed as Equation (2) [32]:

(2) $$\begin{array}{c} E=E^{0}+\frac{RT}{nF}\text{In}\left(\frac{C_{\text{O}}}{C_{\text{R}}}\right), \end{array}$$

(3) $$\begin{array}{c} \eta =E-E_{\text{eq}}, \end{array}$$

where *E* and *E*^0^ are applied potential and standard potential, respectively; *R* is the gas constant; *T* is the absolute temperature; *n* is the number of electrons transferred; *F* is the Faraday constant; and *C*_O_ and *C*_R_ represent the concentrations of oxidized and reduced reagents, respectively [31]. Overpotential is defined as the difference between applied potential (*E*) and equilibrium potential (*E*_eq_) as illustrated in Equation (3). In many cases, the overpotential required to achieve a current density of 10 mA cm^−2^ is employed as an important reference to assess electrocatalysts [34].

### 2.3. Tafel Slope (*b*) and Exchange Current Density (*j*_0_)

The Tafel slope (*b*) is another important parameter to analyze the kinetics and the reaction mechanism of the OER process. The relationship between overpotential (*η*) and current density (*j*) can be illustrated through the Tafel equation (Equation (4)), in which *b* is the Tafel slope. The linearly fitted Tafel slope can be described as “how fast the current increases against overpotential” [41]. Additionally, exchange current density (*j*_0_), which shows the electrocatalyst's catalytic activity at equilibrium status, can be calculated through the Tafel equation by assuming an overpotential of 0. An ideal electrocatalyst should have a small Tafel slope (*b*) and a high exchange current density (*j*_0_).

(4) $$\begin{array}{c} \eta =a+b\log\left(j\right). \end{array}$$

### 2.4. Turnover Frequency (TOF)

Evaluating the overpotential or the Tafel slope values of a given electrocatalyst is not a perfect method to judge its overall catalytic performance due to differences in loading mass [6]. Turnover frequency (TOF), which is defined as the number of reactant molecules transformed per catalytic site over a unit of time, has been proposed to address this problem. TOF can be calculated through equation (5):

(5) $$\begin{array}{c} \text{TOF}=\frac{jA}{zFn}. \end{array}$$

Here, *j* is the current density, *A* is the working area, *z* is the number of transferred electrons, *F* is the Faraday constant, and *n* is the mole amount. However, it should be noted that a precise TOF value is very difficult to obtain because not all active atoms are involved in a real catalytic reaction, so the mole amount *n* in this equation cannot be precisely calculated [34].

## 3. Synthesis Methods for Self-Supported LDH

To synthesize self-supported LDHs with excellent OER activity, both the substrates and synthesis methods should be taken into consideration. Typically, a substrate not only can serve as a current collector for LDH growth but can also provide a tremendous number of diffusion pathways for the release of oxygen bubbles. Conductive substrates like nickel foam (NF), copper foam (CF), carbon cloth (CC), and carbon paper (CP) are usually chosen to grow LDHs because of their low cost, good conductivity, and excellent stability in alkaline electrolyte. During the last few years, a variety of fabrication techniques has been explored to synthesize self-supported LDH electrocatalysts with specific structures and morphologies. Here, the three most-used methods (hydrothermal reaction, electrodeposition, and ion exchange) are introduced to provide a comparative overview of self-supported LDH fabrication. A few other methods like etching and coprecipitation, which are not often seen in the literature, will be introduced during the discussion of specific self-supported LDH electrocatalysts.

### 3.1. Hydrothermal Reaction

Synthesizing self-supported LDHs through a hydrothermal reaction is thought to be the most advantageous method due to its facile operation process and simple chemical reaction [42]. A hydrothermal reaction is carried out by mixing metal salts and precipitating agents together into a solvent and subjecting the solution to thermal treatment under a specified temperature. A precipitator like urea or ammonia plays a significant role in this method because the OH^−^ anions provided by the precipitator can attract metal ions to form metal hydroxides in the initial nucleation step and help to form layered structures during the subsequent hydrothermal treatment. Typically, the self-supported LDH-based electrocatalysts obtained through hydrothermal reaction are homogeneously ordered nanoplates or nanorods with excellent crystallinity and relatively large surface areas [43]. The morphology and structure of self-supported LDHs obtained *via* hydrothermal reaction can be easily controlled by adjusting the reaction parameters, such as temperature, pressure, and reaction time. Moreover, dopants and composites could be introduced into the as-obtained self-supported LDHs through a second hydrothermal reaction. However, it should be noted that the chemical composition and growth rate of LDHs fabricated *via* hydrothermal reaction cannot be precisely controlled because of the sealed reaction environment employed and the long thermal-treatment time required.

### 3.2. Electrodeposition

The electrodeposition method is often applied to fabricate self-supported LDH films on conductive substrates in a two- or three-electrode configuration. The substrate is employed as the working electrode while an aqueous solution containing metal salts acts as the electrolyte in this system. The reduction reactions between the metal ions and the OH^−^ lead to the formation of a self-supported LDH nanoarray on the substrate. This simple and time-saving process can be finished within a quite short amount of time ranging from several seconds to several minutes. The thickness and crystallinity of the LDH can be adjusted by changing the deposition parameters, such as the current density and working time, while its chemical composition can be controlled by changing the metal salts in the electrolyte.

### 3.3. Ion Exchange

Ion exchange is generally conducted to fabricate self-supported LDHs *via* replacing the ions of the host material with other high-mobility ions in the solvent. When employed to synthesize a self-supported trimetallic LDH, the ion-exchange method can introduce new cations into the precursor bimetallic LDH while inheriting its morphology and microstructure. For example, Wang et al. dipped an as-prepared NiCo LDH precursor into an iron nitrate aqueous solution to substitute Fe^3+^ for Ni^2+^ and Co^2+^. Various types of NiCoFe LDHs with different amounts of iron could thus be obtained by changing the iron nitrate content in the solvent [44].

## 4. Self-Supported LDH Materials as OER Electrocatalysts

Non-noble-metal-based LDH materials have emerged as competitive OER electrocatalysts due to their low cost and high electrocatalytic activity. In particular, LDH nanohybrids directly grown on conductive substrates, forming self-supported LDHs, are believed to have advantages such as enhanced electron transport, enhanced electrolyte penetration, and abundant active sites for OER. This type of promising electrocatalyst has increasingly attracted attention among researchers, and many related studies have been published. Here, we will provide a brief overview on recent progress in self-supported bimetallic and trimetallic LDHs, as well as their derivatives, toward OER.

### 4.1. Self-Supported Ni-Based Bimetallic LDH

**Figure 1:**
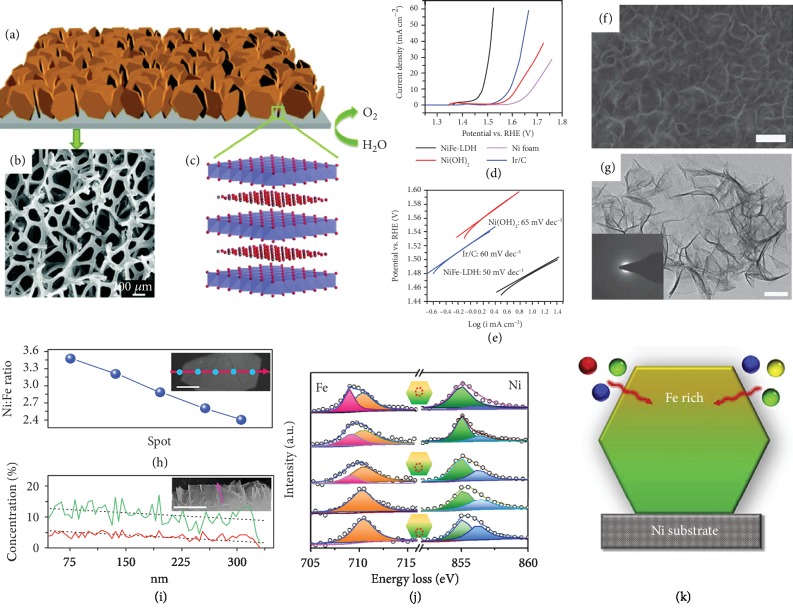
(a) Schematic illustration of NiFe LDH nanoplates grown on NF, (b) SEM image of pure NF, (c) crystal structure of NiFe LDH, (d) polarization curves of different electrocatalysts, and (e) Tafel plots of NiFe LDH/NF (black), Ni(OH)_2_/NF (red), and 20 wt% Ir/C (blue) catalysts in a 1 M KOH solution [47]. Copyright 2014, the Royal Society of Chemistry. (f) High-resolution SEM image of 3D amorphous NiFe LDH/NF (scale bar: 200 nm) and (g) TEM image of NiFe LDH nanosheets scratched off from the NiFe LDH/NF and (inset) corresponding selected area diffraction pattern (scale bar: 10 nm) [49]. Copyright 2015, Nature Publishing Group. (h) TEM line scan and (inset) TEM image of gradient NiFe LDH (scale bar: 100 nm), (i) SEM line scan and (inset) SEM image of gradient NiFe LDH (scale bar: 500 nm), (j) EELS spectra of the Fe edge and Ni edge in gradient NiFe LDH, and (k) illustration of gradient NiFe LDH prepared by the hydrothermal method [51]. Copyright 2019, Elsevier Ltd.

Among all of the self-supported bimetallic LDH catalysts reported thus far, nickel- (Ni-) based LDHs have been studied the most. The fabrication of NiFe LDH was achieved in early studies, but its application for OER was barely studied until Gong et al. first used the solvothermal method to synthesize crystalline NiFe LDH nanoplates on carbon nanotubes as OER electrocatalysis in 2013 [45]. Following this achievement, quite a few studies have focused on this kind of highly efficient electrocatalyst for water oxidation. In particular, to conquer the drawback of low conductivity associated with powdery NiFe LDH, constructing three-dimensional (3D) NiFe LDH on a substrate is highly desirable [46]. For instance, Lu et al. employed Ni(NO_3_)_2_·6H_2_O and Fe(NO_3_)_3_·9H_2_O as metal sources and urea as the precipitant, followed by a hydrothermal reaction at 120°C for 12 h to fabricate 3D NiFe LDH nanoplates on NF (Figures 1(a)–1(c)) [47]. Morphology characterization reveals that these NiFe LDH nanoplates are vertically aligned on the NF as separate intact units. The largest dimension of each individual nanoplate is around 200-300 nm, and the distance between the nanoplates is around 100 nm. Such a mesoporous 3D NiFe LDH/NF electrocatalyst can enhance the electrochemical catalytic reaction by providing a rich source of exposed active sites (Ni and Fe) on the surface, and it shows excellent OER performance with an onset overpotential of 230 mV (Figure 1(d)) and a Tafel slope of 50 mV dec^−1^ in a 1 M KOH solution (Figure 1(e)).

It is generally acknowledged that oxygen bubbles generated during the water-splitting process can attach to the surface of electrocatalyst. They will separate the active sites from the electrolyte and prevent charge/mass transfer, resulting in the incremental increase of overpotential [48]. This phenomenon is especially severe at high current densities, as more oxygen bubbles will be produced. Thus, Lu et al. developed a freestanding NiFe LDH/NF electrode by coelectrodepositing NiFe hydroxides onto a NF substrate [49]. The ratio of Ni : Fe was determined to be 3 : 1 through X-ray photoelectron spectroscopy (XPS) measurements, indicating that the chemical composition of the NiFe LDH is Ni_3_Fe(OH)_9_. Scanning electron microscopy (SEM) and transmission electron microscopy (TEM) images (Figures 1(f) and 1(g), respectively) show the ultrathin amorphous NiFe LDH nanosheets uniformly interconnected across the whole Ni support, forming a special hierarchical structure. The high porosity of this self-supported NiFe LDH/NF electrode could improve the wetting property on the surface and tremendously enhance gas-bubble dissipation. This binder-free NiFe LDH/NF catalyst exhibits an overpotential of 270 mV at current density of 1 A cm^−2^ in 10 M KOH electrolyte. Although the overpotential is low, its base concentration of 10 M is too high to be practical. Its TOF obtained at an overpotential of 400 mV is 0.075 s^−1^, three times higher than that of a commercial Ir/C electrode (0.027 s^−1^) at the same overpotential. Later, Liu et al. successfully synthesized porous aligned single-crystalline NiFe LDH nanoflakes less than 20 nm thick on NF [50]. Such a catalyst exhibits overpotentials of 240 and 260 mV at current densities of 50 and 100 mA cm^−2^ in a 1 M NaOH solution, respectively, as well as a small Tafel slope of 31 mV dec^−1^.

More recently, Zhou et al. applied an asymmetrical gradient effect to a self-supported NiFe LDH through a facile nanoarray construction technique in order to boost its OER performance [51]. TEM line scan and electron energy loss spectroscopy (EELS) measurements indicate that the Ni : Fe concentration ratio of this NiFe LDH nanoarray supported on NF decreases from 3.5 : 1 to 2.4 : 1 from the bottom to the top (Figures 1(h)–1(k)). Meanwhile, both the Ni and Fe in the NiFe LDH nanoarray preferentially remain at the lower valent state due to the reduction effect by Ni foam. Theoretical calculations prove that the gradient effect could enhance the OH^∗^ binding strength to the Ni sites, thus modulating O^∗^ and OOH^∗^ binding to the Fe sites, which can lower the absorption energy and decrease the overpotential required for the OER process. Further XPS characterizations and theoretical calculations reveal that the long-range gradient in NiFe-LDH could accelerate the electron transfer from the top to the bottom by changing the band structure. When utilized in water splitting, the self-supported gradient NiFe LDH/NF nanoarray exhibits an overpotential of 330 mV at 100 mA cm^−2^ in a 1 M KOH solution.

**Figure 2:**
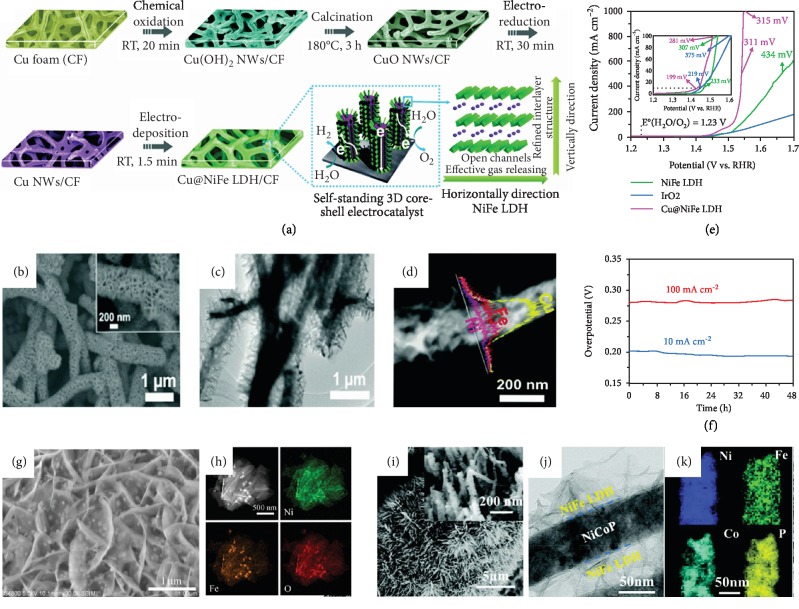
(a) Schematic illustration of the fabrication process for the self-supported 3D core-shell Cu@NiFe LDH electrocatalysts (RT: room temperature), (b) SEM images of Cu nanowires, (c) TEM image of Cu@NiFe LDH, (d) EDS line scan spectrum of Cu@NiFe LDH, (e) polarization curves of Cu@NiFe LDH measured in 1 M KOH, and (f) chronopotentiometry curves of Cu@NiFe LDH at constant current densities of 10 and 100 mA cm^−2^ [35]. Copyright 2018, the Royal Society of Chemistry. (g) Low-magnification FESEM image and (h) elemental mapping images of NiFe_2_O_4_ nanoparticles/NiFe LDH [59]. Copyright 2018, American Chemical Society. (i) SEM image of NiFe LDH@NiCoP/NF and (inset) high-resolution image of its NiFe LDH@NiCoP nanowires and (j) TEM image and (k) EDS mapping of a specific NiFe LDH@NiCoP nanowire [60]. Copyright 2018, Wiley-VCH.

In addition to morphology and structure control, the substrates of self-supported NiFe LDH electrocatalysts also play a vital role in their catalytic performance. Compared with nickel foam, copper foam (CF) has higher electron conductivity and lower cost, which is beneficial for electron transport and large-scale applications. Based on this concept, Yu et al. fabricated a freestanding Cu@NiFe LDH catalyst on CF toward highly efficient OER [35]. As illustrated in Figure 2(a), Cu(OH)_2_ nanowires were initially constructed on CF *via* chemical oxidation and were then transformed to CuO and Cu nanowires *via* further calcination and electroreduction, respectively. Finally, NiFe LDH nanosheets were electrodeposited on these Cu nanowires, leading to the formation of a 3D core-shell Cu@NiFe LDH catalyst (Figures 2(b)–2(d)). The porous 3D core-shell nanoarray structure not only provides a larger surface area and more exposed active sites for the catalytic reaction but also promotes water-molecule adsorption and makes the water-oxygen reaction easier. The direct growth of NiFe LDH nanosheets on CF can decrease the electrical contact resistance between the catalyst layer and the substrate. Electrochemical impedance spectroscopy (EIS) results show that this Cu@NiFe LDH electrode has a small charge-transfer resistance of only 2.8 *Ω*, indicating desirable electron transfer and catalytic kinetics. This catalyst exhibits outstanding OER performance with overpotentials of 311 mV and 315 mV at high current densities of 0.5 A cm^−2^ and 1 A cm^−2^, respectively, as well as a small Tafel slope of 27.8 mV dec^−1^ (Figure 2(e)) in a 1 M KOH solution. Additionally, the original structure (shown in the SEM and TEM images in Figures 2(b) and 2(c), respectively) and chemical composition (verified by elemental mapping through energy-dispersive X-ray spectroscopy (EDS, Figure 2(d))) of this catalyst are well preserved after stability testing at 100 mA cm^−2^ for 24 h, showing the good mechanical stability and potential commercial application of this 3D Cu@NiFe LDH catalyst (Figure 2(f)). Similarly, carbon rods (CR) and nickel iron alloy foam (NIF) have also been developed as substrates to grow self-supported NiFe LDH. Li et al. synthesized NiFe LDH on CR and then expanded the interlayer distances with the assistance of a water bath and ultrasound treatment [40]. The expanded interlayers are thought to provide more space for diffusion of the reactants and bubbles, thus leading to better OER activity. Xie et al. fabricated partially amorphous NiFe LDH nanosheet arrays on NIF *via* a hydrothermal reaction [52]. This unique partially amorphous structure creates Ni^3+^ cations and increases the concentration of high-valence active sites for OER while the NiFe alloy foam with an optimized Ni : Fe ratio is believed to have long-term stability in alkaline media toward OER. From the discussion above, we can see that not only the synthesis methods and specific structures but also the substrates can affect the electrical performance of NiFe LDHs toward water splitting. For example, NF is the most widely employed substrate used to grow self-supported NiFe LDH, while CF can produce a unique 3D core-shell structure to further enlarge the active surface area. On the other hand, substrates like CR and NIF can guarantee the long-term stability of NiFe LDHs in alkaline solutions due to their inert properties. To drive 100 mA cm^−2^ current density in 1 M KOH electrolyte, NiFe/NF LDH [50], Cu@NiFe LDH [53], and NiFe/NIF LDH [52] need overpotentials of 260, 281, and 326 mV, respectively. The LDH materials can be grown on different kinds of substrates as long as the substrates can work in alkaline solutions. To achieve large surface area and good contact between the active material and the electrolyte, substrates with 3D hierarchical structures are favorable.

Designing NiFe LDH into a hierarchical architecture and further combining it with other high-performance nanostructured catalysts is a reasonable way to enhance its OER activity. Chi et al. explored a hydrothermal approach to synthesize vertically aligned FeOOH/NiFe LDHs on NF with an effective distribution of active sites and fast ion/electron transfer [54]. They claimed that the Fe^3+^ in Ni_1−*x*_Fe*_x_*OOH can shorten the distance of the Fe−O bond and create strong electronic contact between Fe and Ni, which can accelerate the catalytic OER activity. Electrochemical measurements show that these self-supported FeOOH/NiFe LDHs have quite low overpotentials of 208 and 288 mV required to achieve current densities of 10 and 500 mA cm^−2^ in a 1 M KOH solution, respectively. Transition-metal selenides are currently being explored as promising candidates for OER catalysts due to their inherently high conductivity and comparable catalytic activity. Hou et al. designed a ternary electrocatalyst composed of NiFe LDH anchored on Co_0.85_Se nanosheets further supported on exfoliated graphene (EG) foil [55]. Among these three components, the NiFe LDH with inherently high OER catalytic activity provides a unique nanoarray structure to construct Co_0.85_Se hybrids, the Co_0.85_Se nanosheets not only provide abundant positively charged Co^2+^ and Co^3+^ active sites to which the OH^−^ in alkaline media can attach but also promote the conductivity of this 3D electrode due to its half-metallic character, and the EG foil ensures strong interaction in the hybrid and enables efficient charge transfer. The intrinsic merits and strong coupling effects of these three components contribute to the higher OER activity and stability of this self-supported EG/Co_0.85_Se/NiFe LDH electrocatalyst. Likewise, Dutta et al. designed a self-supported NiFe LDH-NiSe/NF electrocatalyst through a two-step hydrothermal process [56]. Thin-layer NiFe LDH was deposited onto the as-obtained NiSe*_x_*/NF by heat treatment with an aqueous solution containing iron nitride. In addition to the active sites located on the NiFe LDH layers, the NiSe can provide additional catalytic active sites during the OER process. Meanwhile, the thin lamellar morphology of these two active centers and the porous structure of the resultant catalyst can facilitate charge transfer and electrolyte adsorption. The combination of NiSe and NiFe LDH significantly enhances the OER performance, so that an overpotential of only 240 mV is required to achieve a current density of 10 mA cm^−2^ in 1 M KOH. Li et al. further reported a freestanding cactus-like (Ni,Co)Se_2_ shell with NiFe LDH on CC, in which the surfaces and edges of the nanosheets provide a uniform scaffold for the self-assembled 1D nanospines [57]. These nanospines offer a pathway for effective charge transfer and accelerate the release of oxygen bubbles, while the nanosheets provide good mechanical strength and stability.

Recently, ternary transition-metal sulfides (TMSs), oxides (TMOs), and phosphides (TMPs) have aroused extensive interest as electrocatalysts for water splitting due to their high electrocatalytic activity and good stability [39]. Coupling these ternary transition-metal-based catalysts with NiFe LDH to construct self-supported hierarchical LDH structures is an effective method to enhance the OER activity. For example, Liu et al. combined density functional theory (DFT) calculations with experimental research to design a NiCo_2_S_4_@NiFe LDH electrocatalyst for overall water splitting [58]. The DFT calculations first indicated that the NiCo_2_S_4_ and the NiFe LDH have strong interaction and efficient charge transfer capability at their interface, implying that the combination of these two materials may have a dramatic influence on their catalytic activity. Moreover, the free energy of hydroxide (Δ*E*_OH_) calculated from DFT is reduced from 1.56 eV for pure NiFe LDH to 1.03 eV for the NiCo_2_S_4_@NiFe LDH composite, showing a lower adhesion energy during the OER process. They then successfully synthesized the NiCo_2_S_4_@NiFe LDH catalyst, for which NiCo_2_S_4_ nanotubes were first grown on NF and then coated by NiFe LDH nanosheets. This self-supported electrocatalyst with a heterostructure interface requires an overpotential of only 201 mV to achieve a current density of 60 mA cm^−2^ in a 1 M KOH solution, lower than the overpotentials of NiFe LDH/NF (260 mV) and NiCo_2_S_4_/NF (306 mV) needed to achieve the same current density. Integrating ternary TMOs with NiFe LDH to obtain enhanced catalytic performance was proposed by Wu et al. As shown in Figures 2(g) and 2(h), the NiFe_2_O_4_ nanoparticles are homogenously attached on the NiFe LDH nanosheets, offering a larger active surface area [59]. This NiFe_2_O_4_ nanoparticles/NiFe LDH electrode with optimized electronic and surface structures shows outstanding OER performance with an ultrasmall overpotential of 265 mV needed to achieve a large current density of 1 A cm^−2^ in a 1 M KOH solution. Zhang et al. fabricated a 3D hierarchical heterostructured NiFe LDH@NiCoP/NF electrocatalyst *via* a complicated hydrothermal–phosphorylation–hydrothermal reaction [60]. The electrocatalyst has a well-defined two-phase core-shell structure, where NiFe LDH nanosheets are uniformly anchored on the NiCoP nanorods, as shown in Figures 2(i)–2(k). The shift of the Ni 2p_3/2_ and Co 2p_3/2_ peaks of the NiCoP phase in the XPS spectrum indicates an enhanced electronic connection in the interaction between the layered NiFe LDH nanosheets and the NiCoP nanorods. The special interface engineering and synergistic effects together lead to charge-transfer facilitation and reaction-kinetics enhancement. Moreover, the larger electrochemically active surface area (ECSA) of LDH@NiCoP/NF compared to NiFe LDH/NF and NiCoP/NF guarantees more catalytically active sites involved in the OER at high current density, leading to enhanced OER activity and decreased potential aggregation. The strong connection between the NiCoP nanowires and the NiFe LDH nanosheets leads to synergistic effects toward electrocatalytic reactions and maintenance of good mechanical stability. This hierarchical NiFe LDH@NiCoP/NF electrode shows no obvious degradation after 100 h continuous testing, indicating excellent catalytic activity and long-term stability. A similar result has also been reported by Zhou et al., who enhanced the OER activity of NiFe LDH by fabricating a CoNiP core @NiFe LDH nanoarray shell on NF [61]. The CoNiP@NiFe LDH nanoarray shows remarkable long-term durability over 500 h OER electrolysis testing.

As discussed above, we can conclude that the intensified OER activity of self-supported NiFe LDHs and their derivatives mainly originates from the following: (1) the 3D hierarchical structure of the self-supported NiFe LDH guarantees abundant open spaces, which can accelerate the electrolyte diffusion and promote the rapid release of gas bubbles; (2) the self-supported NiFe LDH on conductive bones has fast electron/mass transport among catalysts and current collectors; and (3) the direct integration of NiFe LDH with other materials results in strong coupling effects, which can create more active sites and lower the charge-transfer resistance. Additionally, the intrinsic merits of the compositions, such as their high electrocatalytic activity and durability, contribute to the enhanced OER activity and stability of NiFe LDH.

**Figure 3:**
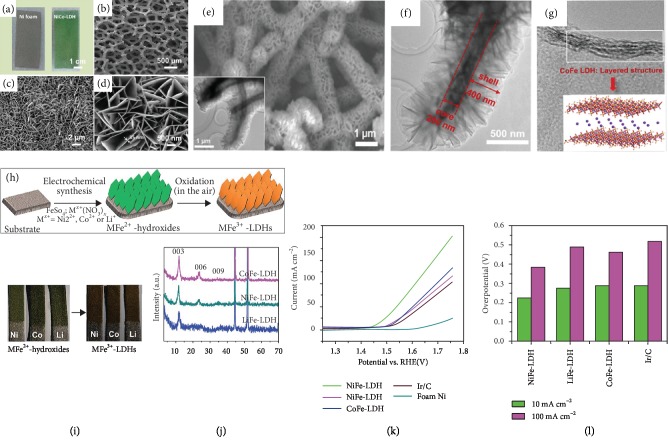
(a) Optical and (b-d) SEM images of NiCo LDH nanosheet arrays on NF [62]. Copyright 2015, Elsevier Ltd. (e) SEM and (inset) corresponding TEM images of CCF LDH, (f) TEM image of CCF LDH at medium magnification, and (g) HRTEM image of CCF LDH showing the layered structure of CoFe LDH and (inset) structure model of CoFe LDH [36]. Copyright 2017, Elsevier Ltd. (h) Schematic illustration of the synthetic route to MFe LDH (M = Co, Ni, or Li) nanoarrays, (i) photographs of MFe LDH nanoarrays growing on Ni foam before and after self-oxidation in air, (j) XRD patterns of MFe LDH, and (k) linear sweep voltammetry (LSV) curves and (l) overpotentials (at 10 and 100 mA cm^−2^ in 1 M KOH) of MFe LDH in comparison with the benchmark Ir/C electrocatalyst [69]. Copyright 2015, the Royal Society of Chemistry.

Some studies have shown that self-supported NiCo LDH exhibits OER activity similar to that of NiFe LDH. Jiang et al. employed a solvothermal process in which methanol serves as both solvent and reactant to grow a 3D network of NiCo LDH nanoarrays on NF (Figures 3(a)–3(d)) [62]. XPS spectra show that Ni and Co in this electrocatalyst exist in multiple valence states (Ni^2+^/Ni^3+^ and Co^2+^/Co^3+^), which are active centers during the water-splitting process. It has been confirmed that Ni and Co in higher valence states exhibit better OER catalytic activity [63, 64]. Furthermore, the dual active sites in NiCo LDH can be *in situ* oxidized to more active NiOOH or CoOOH, which are favorable metal species for OER. The 3D microscopically porous structure of the NiCo LDH/NF electrode, along with its unique redox features of Ni and Co species, make it a superior electrocatalyst for water splitting. Exfoliation treatment is another method to promote the electrocatalytic performance of NiCo LDH toward OER. Liang et al. synthesized NiCo LDH nanoplates on a carbon paper (CP) using a high-temperature and high-pressure hydrothermal reactor [65]. This method can be used to exfoliate NiCo LDH into very thin layers with rich active edge sites and greater surface area, which is beneficial to the contact between the electrode and electrolyte media. The NiCo LDH/CP yields an overpotential of 367 mV required to achieve a current density of 10 mA cm^−2^ and a Tafel slope of 40 mV dec^−1^ in a 1 M KOH solution. Yu et al. reported vertically oriented NiCo LDH nanosheets attached on a carbon fiber paper (CFP) [66]. The conductive substrate CFP not only serves as the support and current collector but also modulates the NiCo LDH assembly by avoiding NiCo LDH aggregation. Meanwhile, the widely spaced and vertically aligned NiCo LDH nanosheets on CFP guarantee long-term stability and create fast paths for the transport of both electrolyte ions and electrons. Much earlier, Chen et al. synthesized NiCo LDH on N-doped graphene (NG) hydrogels, which were then deposited onto NF [67]. The high conductivity of NG-NiCo LDH contributes to good OER activity by offering effective charge and mass transfer.

Besides the tremendous research effort toward self-supported NiFe LDH and NiCo LDH, Li et al. developed a novel SC@NiMn-LDH@G electrode for water splitting, in which graphene (G) coats NiMn LDH nanoarrays on conductive sponge-derived carbon (SC) [68]. The highly porous SC enhances the conductivity of NiCo LDH and facilitates the transport of ions in electrolyte. Meanwhile, the hierarchical, interconnected G on the top can create fast channels for electrons to be easily transferred along the NiCo LDH nanosheets. In addition, the G coating leads to enhanced stability of the composite during long-term operation. This well-designed SC@NiMn-LDH@G electrocatalyst exhibits outstanding OER performance, exhibiting both a low overpotential (220 mV required to achieve a current density of 10 mA cm^−2^) and a low Tafel slope (33 mV dec^−1^) in a 1 M KOH solution.

### 4.2. Self-Supported Co-Based Bimetallic LDH

Various self-supported cobalt- (Co-) based LDH materials have been reported for OER electrocatalysis due to the highly efficient Co active sites though Co is much more expensive than Ni and Fe. In a previous study, Yu et al. electrodeposited 2D CoFe LDH nanosheets onto self-made Cu nanowires/Cu foam to fabricate a novel hierarchical core-shell Cu@CoFe (CCF) LDH nanoarchitecture [36]. Both the thickness and the morphology of the composites could be controlled by the electrodeposition time. SEM and TEM images (Figures 3(e)–3(g)) of the CCF with electrodeposition time of 60 s clearly show that the CoFe LDH nanosheets interconnect with one another, forming a highly porous surface morphology. The unique layered structure of CCF is favorable for the diffusion of water molecules and the release of gas products since it ensures intimate contact between the catalyst and electroactive species. Additionally, the highly conductive core of Cu nanowires not only acts as a pathway for the transfer of electrons but also facilitates the reaction kinetics by reducing the distance for electron transfer. When applied in OER, the overpotential required to achieve a current density of 100 mA cm^−2^ is merely 300 mV in a 1 M KOH solution. The SEM and TEM images, as well as the XPS spectra, of the sample after water-splitting stability testing for 48 h are almost the same as those of the fresh sample before testing, indicating its excellent stability.

Li et al. synthesized a series of ultrathin (8–12 nm in thickness) NiFe, CoFe, and LiFe LDH nanoplatelets on NF through a fast electrochemical synthesis route followed by self-oxidation in air, as shown schematically in Figure 3(h) [69]. Photographs in Figure 3(i) show representative nanoarrays growing on NF before and after self-oxidation in air. The specific chemical compositions were determined by XRD measurements (Figure 3(j)). Among all of these bimetallic LDH nanoarrays, NiFe LDH exhibits the best OER activity, with a low overpotential of 224 mV required to achieve a current density of 10 mA cm^−2^ while CoFe and LiFe LDH require overpotentials of 297 and 288 mV for the same current density in a 1 M KOH solution, respectively (Figures 3(k) and 3(l)). The enhanced intrinsic OER activity of these self-supported LDHs is ascribed to the decreased binding energy of O^∗^ to form oxygen gas in the last step of the water-splitting process based on DFT calculations. Feng et al. employed a coprecipitation approach to synthesize a series of Co*_x_*Fe*_y_* LDHs supported on porous NF by adjusting the mole ratio of the reactants [70]. They found that Co_2_Fe_1_ LDH shows the best OER activity with an overpotential of 300 mV required to achieve a current density of 10 mA cm^−2^ in a 1 M KOH solution.

Other than self-supported CoFe LDH electrocatalysts, some new types of Co-based bimetallic LDHs have also been reported. Li et al. fabricated ZnCo LDH thin film on flexible Ni foil *via* an electrodeposition method [71]. Morphology characterization shows that the LDH nanosheets grown on the Ni foil are densely interconnected, resulting in a special nanowall structure. Besides playing a role as a catalytically active site, the Zn^2+^ ions in CoZn LDH mainly promote the reaction species by accessing the oxidized cobalt ions. The TOF of the ZnCo LDH/Ni foil catalyst is around 1.7 times higher than that of monometallic Co hydroxide (3.56 s^−1^*vs.* 2.07 s^−1^, respectively) and 4 times higher than that of ZnCo LDH powder (0.88 s^−1^) at the same overpotential. Later, You et al. reported an electrocatalyst with a CoFe-Borate coating layered on CoFe LDH nanosheets for OER at near-neutral media (0.1 M K_2_B_4_O_7_ solution) [53].

### 4.3. Self-Supported Trimetallic LDHs

Owing to the high flexibility of cations in LDH layers, various types of active cation centers can be introduced into the LDH structure to modify the active species [27]. Bimetallic-ion-based self-supported LDH materials, like NiFe, NiCo, and CoFe LDHs, as well as compositions incorporating these materials, have shown excellent catalytic activity toward water splitting. The OER performance of these pristine LDH materials can be further enhanced by transforming them into trimetallic LDHs, which can be carried out *via* metal doping or intercalation. In general, the introduction of new cations can regulate the electronic structure of a pristine LDH and produce strong synergetic effects between the doping cations and the host cations, ensuring high electrocatalytic activity in trimetallic self-supported LDHs.

**Figure 4:**
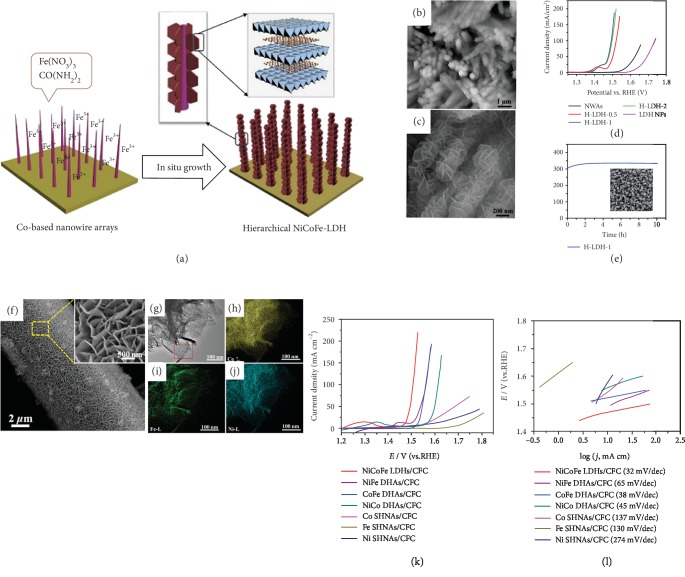
(a) Schematic illustration of the fabrication route to NiCoFe LDHs/NF *via* a two-step hydrothermal reaction, (b, c) SEM images of the hierarchical NiCoFe LDH nanoarrays at different magnifications, (d) polarization curves of different hierarchical LDH electrocatalysts (0.5, 1, and 2 indicate the amount of Fe(NO_3_)_3_·9H_2_O in reactants in mmol), and (e) stability testing of the H-LDH-1 sample under constant potential in a 1 M KOH solution and (inset) SEM image of H-LDH-1 after stability testing [38]. Copyright 2014, the Royal Society of Chemistry. (f) SEM images of NiCoFe LDHs/CFC; (g) HAADF-STEM image of a typical NiCoFe LDH nanosheet and the corresponding STEM-EDS elemental mapping images of (h) Co, (i) Fe, and (j) Ni; and (k) polarization curves and (l) corresponding Tafel curves of NiCoFe LDHs/CFC, NiFe DHNAs/CFC, CoFe DHNAs/CFC, NiCo DHNAs/CFC, Ni SHNAs/CFC, Co SHNAs/CFC, and Fe SHNAs/CFC, where DHNAs and SHNAs are double- and single-hydroxide nanosheet arrays in a 1 M KOH solution, respectively [72]. Copyright 2016, American Chemical Society.

The utilization of self-supported NiCoFe LDHs as OER electrocatalysts was first reported by Yang et al. in 2014 [38]. They developed a two-step hydrothermal method to fabricate trimetallic NiCoFe LDH nanoplates on NF with a controllable size and structure. As the schematic illustration in Figure 4(a) shows, vertically aligned Co_2_(OH)_2_CO_3_ nanowire arrays with diameters of around 90 nm and lengths of around 5 *μ*m were first grown on NF. Ultrathin NiCoFe LDH nanoplates were further grown around these Co-based nanowires *via* a hydrothermal reaction of the as-obtained substrate with an aqueous solution containing iron nitrate and urea. The hierarchically ordered mesoporous structure (Figures 4(b) and 4(c)) not only offers a large surface area for active sites but also possesses a smaller bubble adhesive force. Bubble adhesive force measurements show that these self-supported NiCoFe LDHs/NF catalysts exhibit a low bubble adhesive force of only -2 mN, much smaller than that of the pure NF (-10 mN). Such a small bubble adhesive force guarantees rapid removal of gas bubbles and ensures a constant working area, two key factors in accelerating electrocatalytic performance. In addition, the incorporation of Ni and Fe into Co-based nanowires not only creates new active sites with lower activation energy but also leads to partial-charge transfer between the Ni, Fe, and Co atoms, further enhancing the conductivity of NiCoFe LDHs. Therefore, this electrode exhibits excellent OER performance, with a small overpotential of only 257 mV required to achieve a current density of 80 mA cm^−2^ in a 1 M KOH solution (Figure 4(d)). The structure and morphology of the hierarchical LDH nanoarrays are well retained after 10 h OER testing (inset, Figure 4(e)), indicating good stability. Later, Wang et al. reported a porous structural NiCoFe LDH on flexible carbon fiber cloth (CFC) with excellent physical adhesion and efficient mass transfer [72]. As shown in Figures 4(f)–4(j), the porous networks on the CFC surface are assembled from an enormous number of interconnected NiCoFe LDH nanosheets, where the Ni, Co, and Fe are uniformly dispersed. The well-ordered structure and homogeneous dispersion of cations ensure that the active sites on the catalyst surface can effectively take part in the catalytic reaction and improve the electrode utilization. It is widely accepted that introducing Fe into NiCo LDH can create more active sites through crystal structure disorder and promote conductivity by tuning the electronic structure, leading to superior OER activity. This optimal NiCoFe LDHs/CFC electrode shows much better OER performance than pristine NiCo LDH/CFC, with an overpotential of 280 mV required to achieve a current density of 10 mA cm^−2^ and a Tafel slope of 63 mV dec^−1^ in a 1 M KOH solution (Figures 4(k) and 4(l)).

Beyond the structure control and substrate adjustment of self-supported NiFeCo LDHs mentioned above, Zhu et al. synthesized a series of Ni*_x_*Co*_y_*Fe LDH samples (*x* = 2.5, 2, and 1.5; *y* = 0.5, 1, and 1.5) with different ratios of Ni and Co on NF in order to analyze the effects of the different NiCoFe LDH phases on water splitting [73]. The monolithic-structured Ni_2.5_Co_0.5_Fe/NF LDH exhibits the smallest charge-transfer resistance among all of these binary and ternary LDHs as determined from EIS testing. The decrement of charge-transfer resistance of NiCoFe LDH/NF is attributed to the formation of CoOOH during the water-oxidation reaction, which can accelerate the electron transfer and proton movement. Additionally, optimal Co doping can decrease the activation energy of water oxidation according to the Arrhenius plots, consequently improving the OER activity, while too much Co dopant may result in a weakened synergistic effect and degrade the OER performance. The nonmetallic element sulfur was utilized by Cao et al. to synthesize 3D hierarchical porous sulfur-incorporated NiCoFe LDH nanosheets on carbon cloth (donated as S-NiCoFe/CC LDH) [74]. Inductively coupled plasma-mass spectrometry (ICP-MS) measurements indicate that the exact atomic ratio of Ni : Co : Fe : S in the final production is 2.89 : 0.21 : 1 : 1.3. Such a high proportion of S dopant well maintains the novel hierarchical 2D-layered structure of the NiCoFe LDH and provides a substantial number of active sites. The sulfurization of NiCoFe LDHs could result in a small portion of the hydroxyl ions being substituted by S^2-^, effectively promoting electrical conductivity through the construction of Ni–S and Co–S bonds, which are more favorable for electron transfer. This self-supported S-NiCoFe/CC LDH catalyst shows enhanced electrocatalytic activity toward OER, only requiring a low overpotential of 258 mV to achieve a current density of 100 mA cm^−2^ in a 1 M KOH solution.

**Figure 5:**
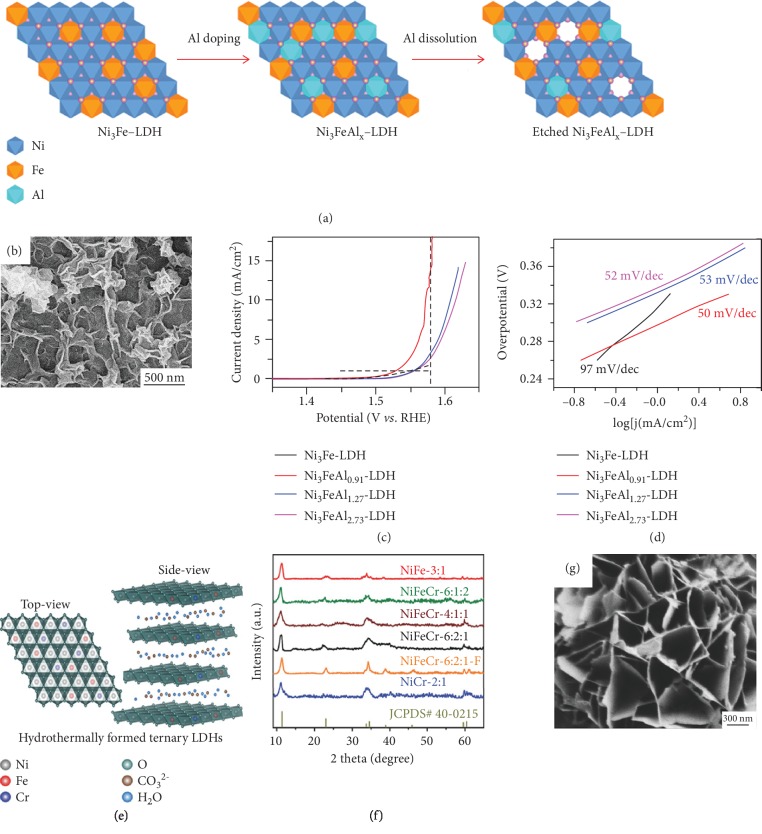
(a) Schematic illustration of the preparation of Ni_3_FeAl*_x_* LDH nanosheets *via* Al doping and selective etching, (b) SEM image of Ni_3_FeAl_0.91_ LDHs/NF, and (c) iR-corrected OER polarization curves and (d) Tafel plots of Ni_3_FeAl*_x_* LDH nanosheets recorded at a scan rate of 10 mV S^−1^ in a 1 M KOH solution [75]. Copyright 2017, Elsevier Ltd. (e) Schematic illustration of the NiFeCr LDH crystal structure with intercalated water and carbonate ions, (f) XRD patterns of as-prepared LDH samples, and (g) SEM image of NiFeCr-6 : 2 : 1-F [77]. Copyright 2018, Wiley-VCH.

Doping combined with etching has been demonstrated as an effective approach to create more active sites and to enlarge the ECSA of self-supported trimetallic LDH catalysts. As a typical example, Liu et al. designed and synthesized Ni_3_FeAl*_x_* LDH (*x* = 0, 0.91, 1.27, and 2.73) nanoplates on porous NF *via* aluminum doping and dissolution (Figures 5(a) and 5(b)) [75]. The introduction of trivalent Al ions into NiFe LDH effectively creates disordered or amorphous structures with abundant defects or vacancies. Such a unique structure can increase the concentration of slightly coordinated Ni and Fe (such as Ni^3+^ and Fe^3+^) active sites on the catalyst surface. Moreover, a great number of defects on the atomic scale are formed by the partial etching/dissolution of Al^3+^ in a KOH solution, which further increases the exposed active edges and enhances the OER activity. The Ni_3_FeAl_0.91_ LDH/NF electrode shows the highest catalytic activity with a Tafel slope of 57 mV dec^−1^ and an overpotential of 304 mV required to achieve a current density of 20 mA cm^−2^ in a 1 M KOH solution (Figures 5(c) and 5(d)). Compared with Al, chromium (Cr) has more oxidation states (from +1 to +6), among which Cr^3+^ and Cr^4+^ are common electrocatalytic active sites. Thus, Cr might be a suitable candidate dopant to promote the OER performance of NiFe LDH. Bo et al. employed an electrochemical deposition-etching procedure to fabricate holey-structured NiFeCr LDH on NF (h-NiFeCr/NF) [76]. The chemical composition of the h-NiFeCr LDH was determined by XPS analysis to be Ni_3_FeCr(OH)_12_. In this h-NiFeCr/NF catalyst, unstable electrodeposited Cr cations will dissolve in the alkaline electrolyte, forming a porous structure on the 2D NiFe LDH nanostructures, while the remaining Cr cations can synergistically modulate the electronic structure. The enlarged ECSA and enhanced conductivity of the h-NiFeCr/NF electrode generated from Cr doping and etching are beneficial to its OER performance. Moreover, the development of such an electrode suggests that developing electrolyte/gas holes through a doping and etching method would be a facile and reasonable way to design porous structured self-supported trimetallic LDH catalysts. Similarly, Yang et al. designed a series of trimetallic NiFeCr LDHs on carbon paper (CP) electrocatalysts for improved OER activity (Figures 5(e) and 5(f)) [77]. In this work, they explained how Cr interacts with Ni or Fe and influences the electronic structure of self-supported NiFeCr LDHs/NF. Typically, Cr^3+^, Cr^4+^, and even Cr^5+^ oxidation state cations generated from the introduction of Cr can buffer the multielectron process toward OER. Additionally, Cr dopant can create more redox-active cations with high oxidation states (Ni^3+^ and Ni^4+^; Fe^3+^ and Fe^4+^), which are considered as active sites in the water-splitting process. Finally, the strongly synergistic electron interactions between the multivalent cations (Ni, Fe, and Cr) and the metal hydroxide matrix can also enhance the catalytic activity. Electrochemical testing showed that the composition with the Ni : Fe : Cr molar ratio of 6 : 2 : 1 exhibits the best intrinsic OER catalytic activity among the NiFeCr LDH composites. The authors also proved that F^−^ ions can be introduced into these self-supported NiFeCr LDHs by NH_4_F during the fabrication process to improve the nanostructure morphology and enhance the crystallinity (Figure 5(g)). Iridium (Ir) doping was applied by Chen et al. to promote the poor intrinsic catalytic activity of NiFe LDH by boosting the dissolution kinetics process of water molecules in the water-splitting process [78].

Li et al. chose vanadium (V) as a dopant to enhance the OER performance of NiFe LDH [79]. The incorporation of V into the NiFe LDH layers not only replaces a portion of the Fe sites but also optimizes the chemical environment of the Fe cations, promoting the intrinsic activity. Density functional theory plus U (DFT + U) calculations confirm that V in the NiFe LDH laminate will lower the Gibbs free energy to drive water oxidation, leading to higher electronic conductivity. When the mole ratio of Ni^2+^ : Fe^3+^ : V^3+^ is 6 : 1 : 1, the self-supported NiFeV/NF LDH exhibits the best OER activity observed, with a Tafel slope of only 42 mV dec^−1^ and an overpotential of 195 mV required to achieve a current density of 20 mA cm^−2^ in a 1 M KOH solution. More recently, self-supported CoMoV LDHs on NF were also employed as efficient OER electrocatalysts by Bao et al. [80]. The doping of Mo and V into the Co-based LDH maintained the intrinsic-layered structure of the LDH and further adjusted its electronic structure, leading to better OER activity. In general, doping of high valence state cations like Cr, Ir, and V can effectively create more active sites and tune the electronic structure of bimetallic LDH, which can be a facile way to synthesize innovative trimetallic LDH catalysts.

Defect construction on the atomic scale is another efficient way to accelerate the OER activity of self-supported trimetallic LDH catalysts. Xie et al. introduced Zn and Al sites into NiFe LDH to synthesize defect-rich NiFeZn and NiFeAl LDHs on NF by selective etching [81]. DFT + U computations demonstrate that the generation of dangling Ni–Fe sites by defect construction of Ni–O–Fe sites lowers the Gibbs free energy of the OER process. Moreover, the deprotonation step in the oxygen evolution process can be promoted due to the generation of the atomic-scale defects in NiFe LDH, which can create abundant highly active defect sites and enhance the synergetic effect among metal cations.

Based on the analysis above, we conclude that the enhanced electrocatalytic OER performance of the self-supported trimetallic LDHs is mainly due to the following: (1) the introduction of a third type of cation normally maintains the original structure but greatly increases the number of active sites on the surface of trimetallic LDHs for OER compared with the pristine bimetallic LDH; (2) the dopants, especially high valence cations, can dramatically increase the concentration of slightly coordinated atoms in bimetallic LDH, which are reported to be beneficial for OER activity; and (3) doping, along with etching, could create electrolyte-/gas-permeable holes and abundant defect active sites on bimetallic LDH nanostructures, contributing to a tunable electronic structure and a large ECSA. It should be noted that although various kinds of composites and cations have been explored to enhance the OER performance of self-supported LDHs, the exact influence of these composites and cations on the OER activity of these combined or trimetallic LDH electrocatalysts remains unclear. Further studies should be focused on investigating the role of extraneous compositions and dopants and determining how they contribute to the high OER activity.

## 5. Conclusion and Perspective

Efficient OER electrocatalysts are in high demand for lowering the activation energy in order to improve the sluggish kinetics during the water-splitting process. Designing innovative nonnoble metal electrocatalysts with high catalytic activity and long-term durability remains a significant challenge. Self-supported LDHs with the merits of abundant active sites, enhanced adsorption ability of oxygen intermediates, and facile synthesis have emerged as among the most promising OER catalysts. In this review, we provided a summary of the progress made thus far on the design and synthesis of self-supported LDHs for electrochemical water oxidation. Different kinds of bimetallic and trimetallic self-supported LDHs, as well as their derivatives, were summarized, and some typical examples are listed in Table 1. Recent advances in their fabrication control, structural design, and mechanistic analysis, along with strategies for electrocatalytic improvement, were described in order to provide insight into the current state of the art. Despite the progress made thus far, more research effort should be devoted to the following in order to design self-supported LDHs with superior OER activity:

1. Developing novel synthesis methods and finding new substrates are reasonable ways to synthesize efficient self-supported LDH electrocatalysts. Different fabrication techniques result in diverse morphology and structures. The above-mentioned fabrication methods each have intrinsic advantages and drawbacks. Novel methods should be taken into consideration to fabricate new types of self-supported LDHs with controllable structures while retaining merits such as large electrochemically active surface areas and abundant exposed active sites that result from traditional synthesis methods. Currently, the conductive backbones generally employed in lab settings are nickel foam, copper foam, and carbon cloth, which are easy to use in growing specific nanostructures, while stainless steel is more favorable in industrial applications due to its low cost and long-term stability. It would be more reasonable to find new substrates for experimental studies rather than continue to employ the current lab substrates in order to fulfill commercial needs
2. The weak understanding of the oxygen intermediates has become an obstacle that seriously hinders the design of efficient self-supported LDH catalysts. It is essential to understand the catalytic mechanisms of self-supported LDHs at the atomic level through the combination of practical experiments and theoretical analysis. For instance, operando characterization technologies such as *in situ* X-ray absorption spectroscopy (XAS) and *in situ* Raman spectroscopy would help to detect OER intermediates under real working conditions and lead to further insights into what is actually happening throughout the entire water-splitting process. Theoretical calculations like DFT computation can provide researchers with fundamental analysis of the catalytic activity. It would be much more helpful if DFT calculation or other theoretical methods could predict potentially outstanding catalysts for experimentalists to synthesize, which could reduce much of the effort currently needed for trial and error
3. The practical industrial application of self-supported LDHs in water splitting remains a challenging task. Although the OER activity of self-supported LDHs has been dramatically enhanced *via* various kinds of techniques in recent years, the catalytic performance at large current density remains far too low for practical application. Additionally, the irreversible side reactions that occur during long-term water splitting will further lead to “electrocatalyst poisoning” and hinder practical industrial application. Finally, catalysts in the lab setting are mainly fabricated on the microscale. Economic expense and mechanical stability must be taken into consideration when scaling up these self-supported LDHs to exhibit high catalytic activity for industrial production. Each element used in the catalysts must be earth abundant so that the price will not be too high when large quantities are going to be used

## Figures and Tables

**Table 1:** Brief summary of self-supported LDHs toward OER performance.

Electrocatalysts	Substrate^∗^	Overpotential (mV) at current densities (mA cm^−2^)	Tafel slope (mV dec^−1^)	Electrolyte	Ref.
3D NiFe LDH nanoplates	NF	280@30	50	1 M KOH	[47]
3D amorphous NiFe LDH	NF	270@1000	32	10 M KOH	[49]
Single-crystalline NiFe LDH	NF	260@100	31	1 M NaOH	[50]
Gradient NiFe LDH	NF	330@100	41.3	1 M KOH	[51]
3D core-shell NiFe LDH	CF	315@1000	27.8	1 M KOH	[35]
Intercalated NiFe LDH	CR	203@10	42	1 M KOH	[40]
Partially amorphous NiFe LDH	NIF	326@100	38	1 M KOH	[52]
Vertically aligned FeOOH/NiFe LDH	NF	247@100	42	1 M KOH	[55]
Hierarchical NiCo_2_S_4_@NiFe LDH	NF	201@60	46.3	1 M KOH	[58]
Cactuslike structural (Ni,Co)Se_2_/NiFe	CC	205@10	61	1 M KOH	[57]
NiFe_2_O_4_ nanoparticles/NiFe LDH	NF	265@1000	28.2	1 M KOH	[59]
NiFe LDH@NiCoP	NF	220@10	48.6	1 M KOH	[60]
NiCo LDH nanoplates	CP	367@10	40	1 M KOH	[65]
Vertical oriented NiCo LDH	CFP	307@10	64	1 M KOH	[66]
Hierarchical Cu@CoFe LDH	CF	318@100	44.4	1 M KOH	[35]
ZnCo LDH thin film	Ni foil	540@13.3	83	1 M KOH	[71]
NiMn LDH	SC	220@10	33	1 M KOH	[68]
NiCoFe LDHs	CFC	239@10	32	1 M KOH	[72]
Ni_2.5_Co_0.5_Fe LDHs	NF	275@10	52	0.1 M KOH	[73]
Holey NiFeCr LDHs	NF	260@100	29	1 M KOH	[76]
NiFeCr-6 : 2 : 1 LDHs	CP	225@25	69	1 M KOH	[77]
NiFeIr LDHs	NF	200@10	32	1 M KOH	[78]
NiFeV LDHs	NF	195@20	42	1 M KOH	[79]
NiFeZn LDHs	NF	250@105	34.9	1 M KOH	[81]
CoMoV LDHs	NF	270@10	106	1 M KOH	[80]

^∗^NF: nickel foam; CF: copper foam; CR: carbon rods; NIF: nickel iron alloy foam; CC: carbon cloth; CP: carbon paper; CFP: carbon fiber paper; SC: sponge-derived carbon; CFC: carbon fiber cloth.
